# Supplementary material for: Derivation of Oligodendrocyte Precursors from Adult Bone Marrow Stromal Cells for Remyelination Therapy
Source: Cells. 2021 Aug 22;10(8):2166. doi: 10.3390/cells10082166 (PMC8391516; doi:10.3390/cells10082166)
Supplement: Supplementary file 1 [file cells-10-02166-s001.zip › cells-1303759-supplementary.pdf]

Supplementary Figure S1

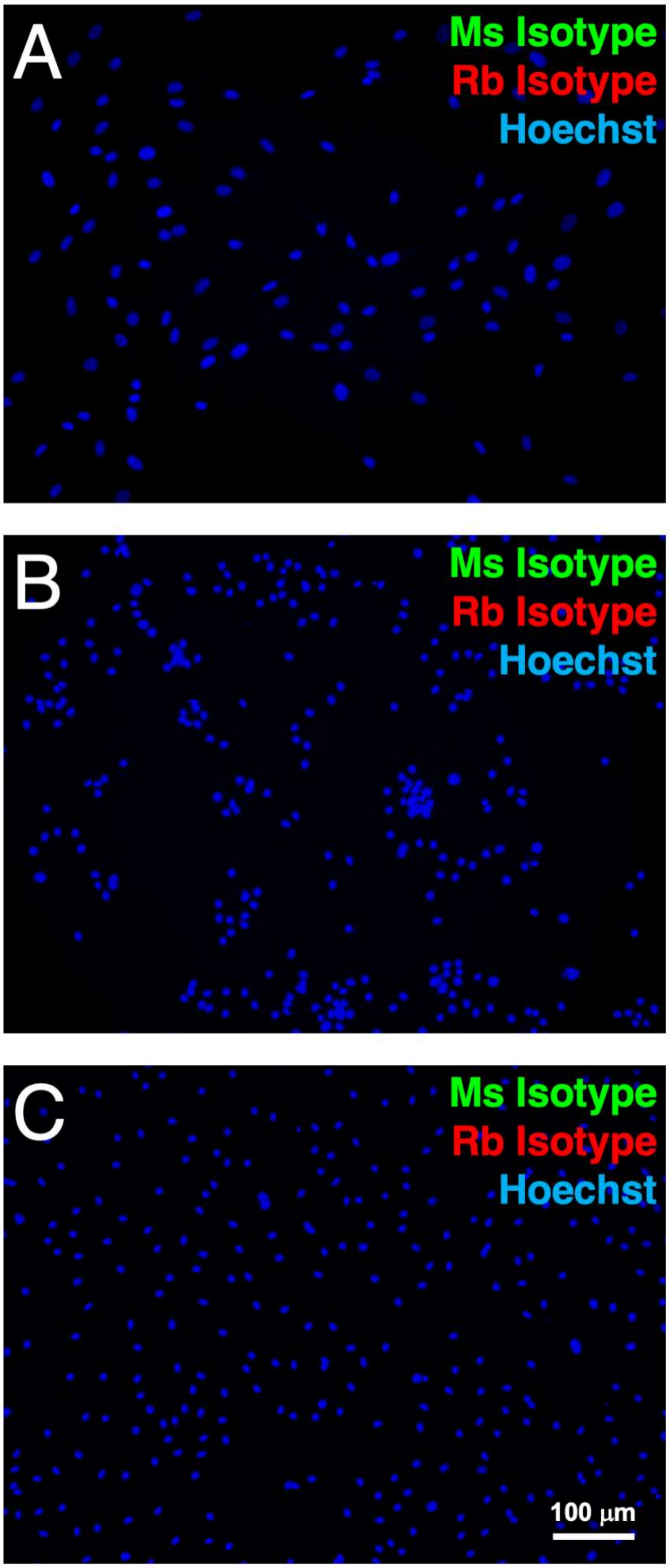

Supplementary Figure S2

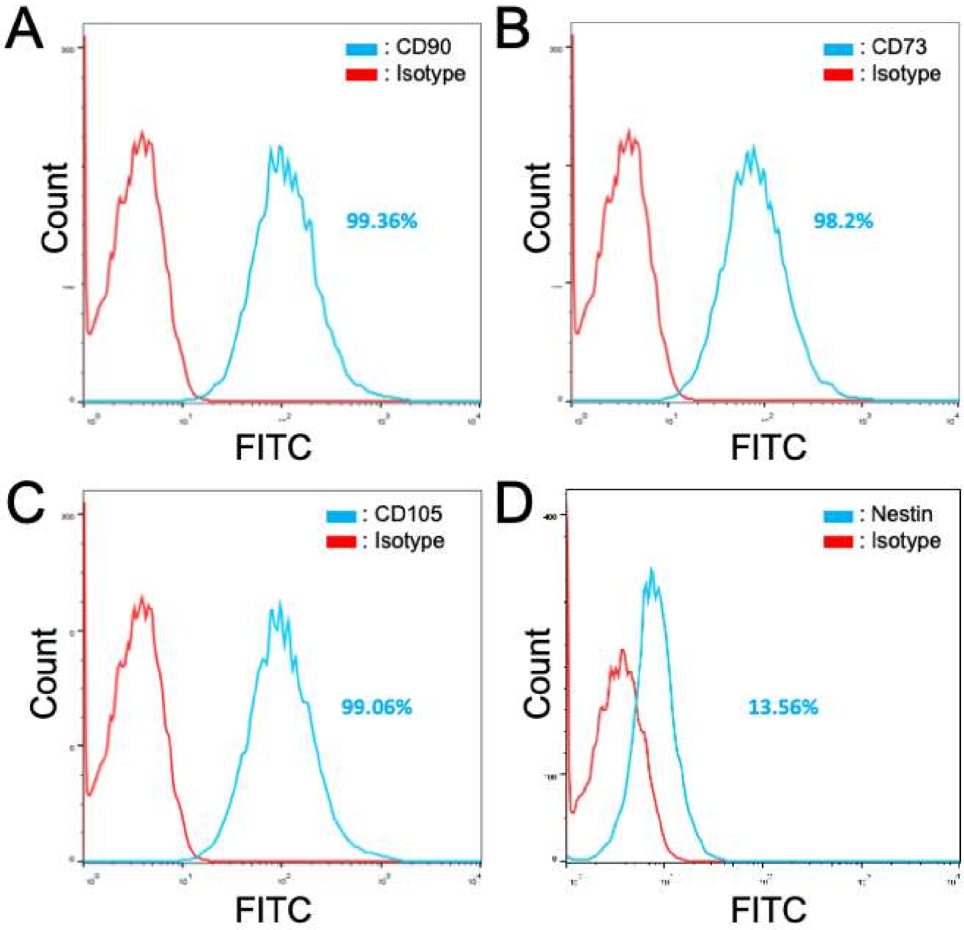

Supplementary Figure S3

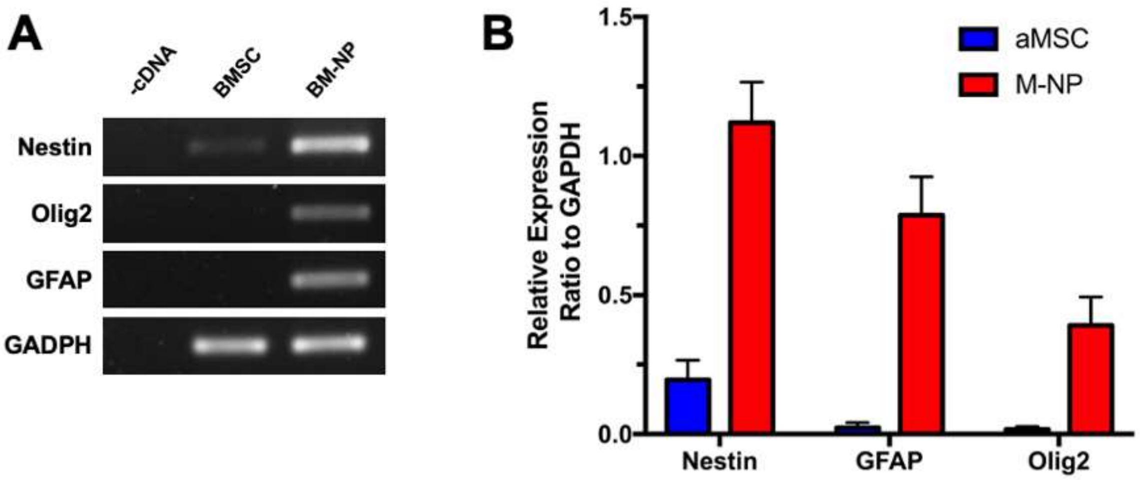

Supplementary Figure S4

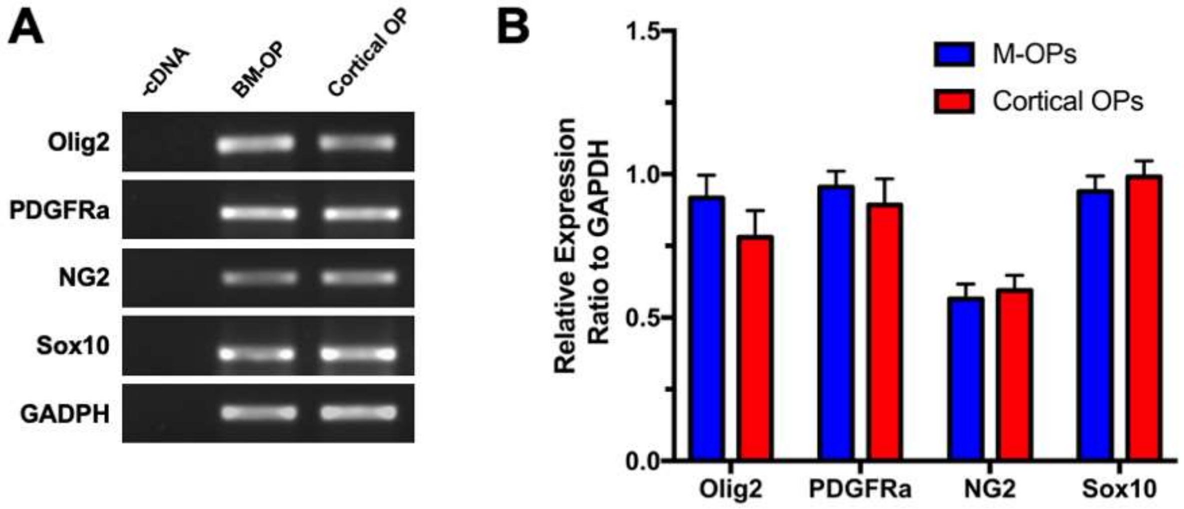

Supplementary Figure S5

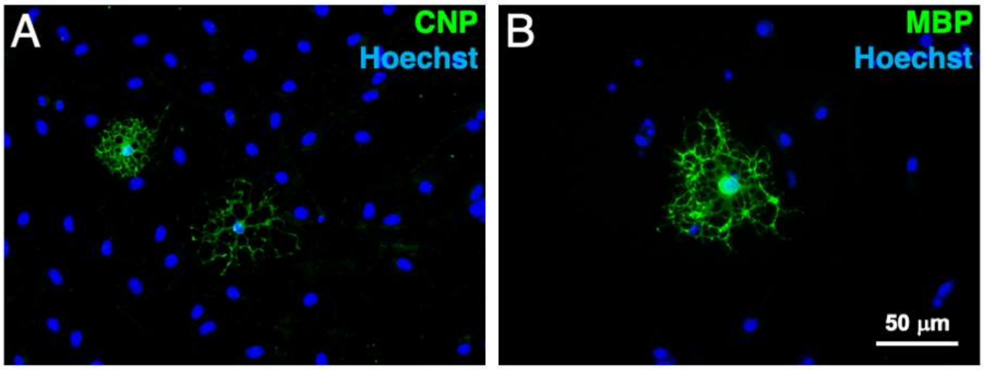

Supplementary Figure S6

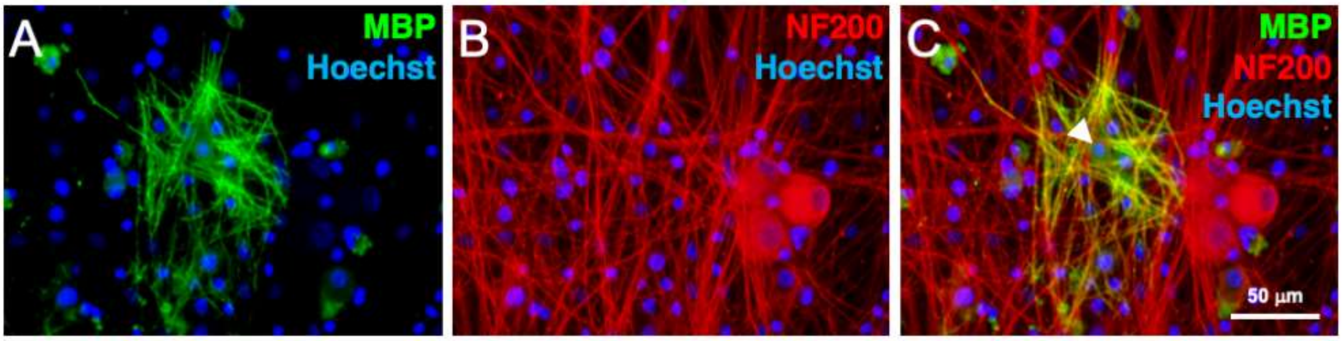

Supplementary Figure S7

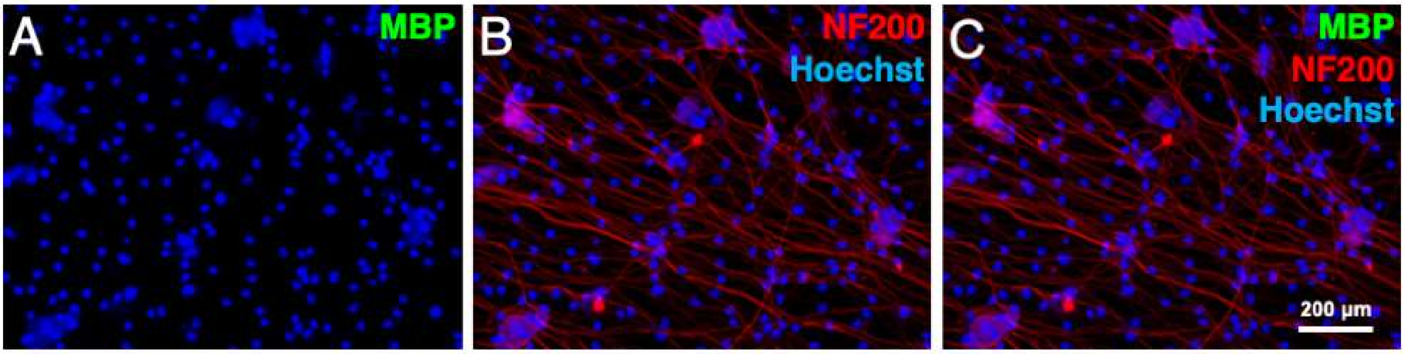

Supplementary Figure S8

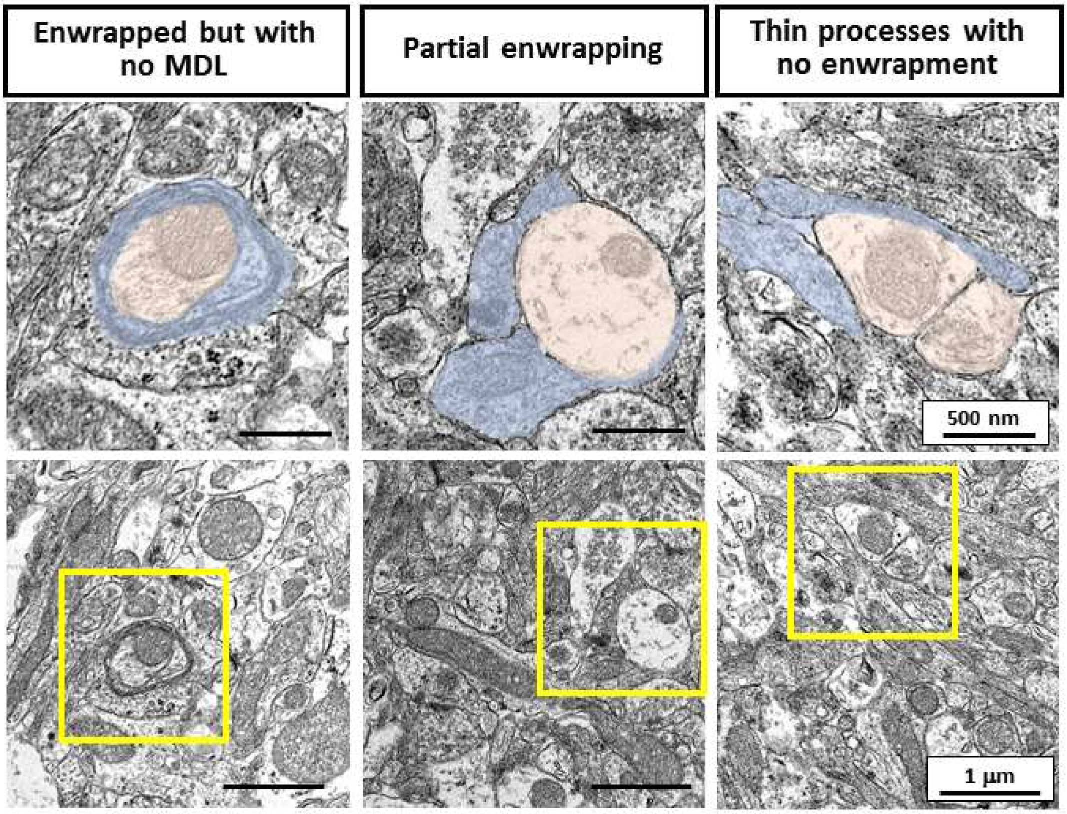

Supplementary Figure Legends

Figure S1: Isotype controls for immunocytochemistry experiments.
Isotype control staining were performed on aMSCs (A), dissociated neurospheres (B) and M-OPs (C). Nonspecific signals were undetectable. n = 4 independent preparations.

Figure S2: Flow cytometry characterization of recovered aMSCs from cryopreservation.
aMSCs recovered from cryopreservation, between passages 3 – 5, were characterized by flow cytometry. Histograms of a representative experiment were shown. Mesenchymal stem cell markers CD90, CD73 and CD105 were found positive in 99.36% (A), 98.2% (B) and 99.06% (C) of cells, respectively. Neural progenitor marker nestin was positive in 13.56% of cells (D). Marker expression profile was not different before and after cryopreservation. n = 4 independent preparations.

Figure S3: Semi-quantitative RT-PCR analysis of aMSC-derived neurospheres.
Expression of nestin, GFAP and Olig2 in neurosphere cultures were compared to those of aMSCs. Nestin expression in neurospheres higher than that of aMSCs, while signals of GFAP and Olig2 were almost undetectable in aMSCs (A). Quantified signal intensities presented in bar chart (B). n = 4 independent preparations. *: p-value < 0.001.

Figure S4: Semi-quantitative RT-PCR analysis of aMSC-derived OPs.
Expression of Olig2, PDGFRα, NG2, and Sox10 in derived M-OPs cultures were comparable to that of primary OP cultures prepared from neonatal rat cortices (A). Quantified signal intensities presented in bar chart (B). n = 4 independent preparations.

Figure S5: Maturation of OP-like cells into OLs after mitogen withdrawal.
CNPase-positive (A) and MBP-positive (B) mature OLs were derived from OP-like cells after mitogen withdrawal. n = 4 independent preparations.

Figure S6: aMSC-derived OPs recovered from cryopreservation generated myelin-forming OLs.
OPs recovered from cryopreservation matured into MBP-positive OLs (A) with process extended along NF200-positive axons (B, merged image in C). OL cell body indicated by white arrow (C). n = 4 independent preparations.
Figure S7: Undifferentiated aMSCs did not generate OLs in co-culture with dorsal root ganglion neurons.

No MBP-positive cells (A) were detected 2 weeks after co-culture of aMSCs and purified dorsal root ganglion neurons (marked by NF200 in B). Merged image is shown in panel C. This indicated that aMSCs were uncapable of generating myelin-forming OLs if the glial induction procedure was omitted. n = 4 independent preparations.

Figure S8: Ultrastructure of myelin in vehicle controls 12 weeks post-transplantation.

Regions boxed in yellow are magnified in the respective upper panels. Majority of OLs (blue) in shiverer mice displayed no/ partial enwrapping of axons (orange) 12 weeks post-injection of medium. Out of more than 1,000 axons that were analyzed in medium-injected shiverer mice, only one OL (left panels) was found to enwrap an axon.

Table S1: Primary antibodies for immunofluorescence

Antibody	Concentration	Manufacturer
Nestin (Mouse IgG)	1:200	BD Pharmingen
GFAP (Rabbit IgG)	1:500	Abcam
Olig2 (Rabbit IgG)	1:400	Abcam
NG2 (Rabbit IgG)	1:200	EMD Millipore
PDGFRa (Rabbit IgG)	1:500	Abcam
Sox10 (Mouse IgG)	1:500	R&D Systems
MBP (Mouse IgG)	1:500	EMD Millipore
NF200 (Rabbit IgG)	1:200	Sigma-Aldrich
RIP (Mouse IgG)	1:200	Hybridoma Bank

Table S2: Primary antibodies for flow cytometry

Antibody	Concentration	Manufacturer
CD90 (Mouse IgG)	1:200	BD Pharmingen
CD73 (Mouse IgG)	1:200	BD Pharmingen
CD105 (Mouse IgM)	1:200	BD Pharmingen
CD45 (Mouse IgG)	1:200	BD Pharmingen
Nestin (Mouse IgG)	1:200	BD Pharmingen
Olig2 (Rabbit IgG)	1:400	Abcam
NG2 (Rabbit IgG)	1:200	EMD Millipore
PDGFRa (Rabbit IgG)	1:500	Abcam
Sox10 (Mouse IgG)	1:500	R&D Systems

Table S3: Primers for RT-PCR

Marker	Forward primer	Reverse primer
Nestin	AACCACAGGAGTGGGAACTG	GGGTCCTCTAGCCCTACCAC
GFAP	TGCCATACAGTGTGAGGGCCTAAA	GCCAAGAACCAAGTTTCACACGCT
Olig2	ACTGATACGGGACTGGATGC	AACACCCCCTCCCAAATAAC
NG2	ACTCTCCCTCCCTGGTGTTTTC	GTCAATGTTTCCATGCTCTGCC
PDGFRa	GAAGGCGCAGAAGCAATAAC	AAACGCGTGGTAAACAGACC
Sox10	AGGGTATGGTGGGAAACCTCAC	GGTGAAAGGATCAGAGTGTCCA
GAPDH	GTC TCA TAG ACA AGA TGG TGA AGG T	TCT TGA GGG AGT TGT CAT ATT TCT

Table S4. Rat MSC proliferation assay

No. of Passage	Time needed to reach ~80% confluence (hr)	MSC Yield (No. of cell per well)	Population Increase (%)
P4	31.17±0.76	7.48±0.42 x 10^5	259.54±14.45
P5	31.33±1.26	7.88±0.43 x 10^5	273.72±14.79
P6	30.67±1.04	8.55±0.52 x 10^5	296.88±18.04
P7	32.33±0.29	8.70±0.59 x 10^5	302.08±20.41
P8	32.83±0.58	8.61±0.27 x 10^5	298.90±9.28

Table S5. OP proliferation assay

No. of Passage	Time needed to reach ~80% confluence	M-OP Yield (No. of cell per well)	Population Increase (%)
P1	71.67±1.26	1.07±0.50 x 10^6	558.59±26.28
P2	73.50±1.80	1.13±0.19 x 10^6	586.46±9.81
